# Peripherally inserted central venous catheter (PICC) in outpatient and inpatient oncological treatment

**DOI:** 10.1007/s00520-019-05276-0

**Published:** 2020-01-22

**Authors:** Dorothea Mielke, Andrea Wittig, Ulf Teichgräber

**Affiliations:** 1 Department of Radiation Oncology, Universitätsklinikum Jena, Bachstr.18, 07743 Jena, Germany and SRH Wald-Klinikum Gera GmbH Straße des Friedens 122, 07548 Gera, Germany; 2 Department of Radiation Oncology, Universitätsklinikum Jena, Bachstr.18, 07743 Jena, Germany; 3 Institut für Diagnostische und Interventionelle Radiologie, Universitätsklinikum Jena, Am Klinikum 1, 07747 Jena, Germany

**Keywords:** Radio-oncology, PICC, Complications, Outpatient and inpatient

## Abstract

**Purpose:**

So far there is little evidence on peripherally inserted central venous catheter (PICC) in radiation oncology patients maintaining the access during the periods of ambulatory and hospital treatment.

**Methods:**

A total of 522 PICC placements in 484 patients were performed between 11/2011 and 07/2016 at the Department of Radiation Oncology and analysed retrospectively for complications and treatment- and patient-related factors during ambulatory and hospital inpatient use. On initial hospitalization, all patients received a multimodal radio-oncological treatment consisting of radiation and intravenous therapy administered via the PICC.

**Results:**

A total of 18,292 catheter days were documented. Median follow-up from catheter insertion to their removal was 37 days (1–97). The overall complication rate was 4.1 per 1000 catheter days (*n* = 75, 14.4%). Complications were similar between the cohort of outpatient 3.6 per 1000 catheter days and the cohort of inpatient 4.8 per 1000 catheter days (OR 0.976; 95% CI [0.598; 1.619]; *p* = 0.924). Severe bloodstream infections occurred at a rate of 0.60 per 1000 catheter days (*n* = 11, 2.1%), deep vein thrombosis at a rate of 0.82 per 1.000 catheter days (*n* = 15, 2.9%) and local inflammation at a rate of 1.26 per 1.000 catheter days (*n* = 23, 4.4%). Only immunotherapy could be identified as an independent risk factor for complications (OR 5.6; 95% CI [2.4; 13.1]; *p* < 0.001).

**Conclusion:**

Using PICC in outpatients is not associated with an elevated risk of complications. Particular attention should be payed to early identification of PICC associated bloodstream infections. Immunotherapy is an independent risk factor for local skin complication.

**Electronic supplementary material:**

The online version of this article (10.1007/s00520-019-05276-0) contains supplementary material, which is available to authorized users.

## Introduction

Peripherally inserted central catheters (PICCs) are non-tunnelled central venous access devices designed for intermediate to long-term use, which are usually placed via a peripheral upper arm vein (i.e. basilica, brachial or cephalic vein) with the catheter tip placed at the cava arterial junction. The average time in situ ranges between 1 week and 6 months; longevity of up to 1 year has been described [1–3]. The technique of a peripherally inserted central venous catheter dates back to the 1970s first used for the parenteral feeding [4]. First attempts were originally accompanied by many complications [5]. Due to medical und technological progress, many of the initial problems have been solved, although some of them (i.e. thrombosis, blood stream infections, etc.) are yet present. The use of PICC has been adequately studied in ICU patients and paediatrics [1, 3, 6–8].

Cancer patients receiving therapy often require an appropriate central venous access to administer chemotherapy, immunotherapy and also for parenteral nutrition, haemodynamic monitoring, blood sampling and supportive therapy [9–11]. Several types of central venous catheter are available at present depending on indication, medical and patient needs. The totally implantable central venous catheter (port catheters) provides long-term (months to years) intermittent access; thin-lumen, non-tunnelled access devices (i.e. central venous catheters (CVC)) are intended only for the short-term use (< 14 days) in hospitalized patients [12, 13]. PICC has been established preferably as medium-term access [2, 3, 13–15].

As a result of medical progress, more and more therapeutic procedures can be performed on an outpatient basis. Moreover, there is an overt political intent to maximize the utilization of outpatient health services because of efficiency in all areas of medicine.

In this context, there is a demand for a safe, reliable, cost-effective and easy-to-use central venous access in cancer patient that can be universally used in both the hospital and community settings.

It is not clear whether PICC meets mentioned requirements and whether it is a favourable alternative to repeated CVC insertion or a more invasive port implantation. Indeed, the evidence for PICC in patients undergoing radio-oncological treatment is low. No reliable clinical data are available on safety of PICC in cancer patient switching from in- to outpatient care during multimodal radiotherapy.

## Objectives

The aim of this study was to investigate practical feasibility and safety profile of PICC in cancer patients receiving radiotherapy in out- and inpatient settings.

### Patients and methods

A total of 522 PICC placements in 484 patients were performed between 11/2011 and 07/2016 at the Department of Radiation Oncology of the Territory University Hospital Jena, Germany, and analysed retrospectively for complications and treatment- and patient-related factors during ambulatory and hospital inpatient use.

The PICC insertion must have been referred by an experienced medical specialist in Radiation Oncology to the Interventional Radiology Vascular Access Service. No cases of foreign PICC indication or insertion were included.

All cancer patients > 18 years were enrolled if PICC was successfully placed for chemotherapy, immunotherapy, total parenteral nutrition, antibiotics or blood sampling.

A total of 545 PICCs signed up for insertion were eligible for the initial selection.

About 23 cases (4.2%) were excluded because of cancelled PICC placement due to lack of patient’s informed consent, wrong medical indication due to, e.g. already existing central venous port system, missing medical follow-up documentation or technical placement failure.

### PICC insertion and maintenance

All PICC insertions were performed by an interventional radiologist following evidence-based institutional protocols (Institute for Diagnostic and Interventional Radiology at University Hospital of Jena) with maximal barrier and antisepsis precautions.

Prior applying a tourniquet, a preprocedural ultrasound evaluation of the potential access site was carried out to identify surrounding anatomical structures, to find an appropriately-sized vessel and to ensure its patency. To minimize the risk of PICC-associated venous thrombosis, the catheter-to-vein ratio of 45% or less should be warranted [16]. We implanted only double lumen 5–6 French PICCs (Bard Access Systems, Salt Lake City, USA; Navilyst Medical, Marlborough, USA). The placement of the catheter at the upper mid-arm into the V. basilica or V. brachialis was performed “real-time” ultrasound-guided using the Seldinger technique under local anaesthesia [15]. The right-sided insertion was preferred unless of medical contraindications.

A small needle was used accessing the target vein followed by introduction of a soft-tipped guidewire to determine the necessary PICC length. The needle was then removed and peel-away sheath was inserted over the guidewire. After the guidewire and dilator were removed, the catheter was threaded through introducer under fluoroscopic control to final tip location at the cavoatrial junction [14, 17].

Fixation to the skin was performed in all patients with sutureless devices (e.g. StatLock® Catheter Stabilization Device, Bard Access Systems) and chlorhexidine-impregnated transparent waterproof films (e.g. Tegaderm™ CHG, 3 M Deutschland GmbH) due to our hospital standard operating procedures (Image 1).

Maintenance and drug delivery were accomplished by trained medical personnel in accordance with hygiene recommendations [2]. Nursing protocol for PICC care included redressing of the catheter exit site with an aseptic technique, flushing and locking of PICCs with prefilled 10 ml normal saline syringes by pulsatile method before and after every intravenous drug delivery

[18, 19]. In both in- and outpatients, PICC maintenance was performed at weekly intervals.

In hospitalized patients, PICC was visually inspected for possible complications at every drug delivery. Ambulant patients got weekly PICC care in our outpatient department. No heparin locking was used, as it is associated with significant risks and no apparent benefit [18].

### Time course of radiation therapy and PICC usage

The PICC insertion was usually carried out as outpatient procedure at the Institute for Diagnostic and Interventional Radiology at Jena University Hospital.

Shortly thereafter patients were hospitalized for initiation of the multimodal radio-oncological treatment. Chemotherapy, immunotherapy, parenteral nutrition or a combination of these was usually administered there via the PICC.

Subsequently patients were discharged and received the rest of radiation therapy on outpatient basis with the PICC still in situ. This venous access was then used for regular blood draws, drug administration and parenteral nutrition.

Readmissions were mostly planned for the next course of chemotherapy but also unplanned because of PICC or oncological complications.

This described switch between outpatient and inpatient treatment happened up to six times (median, 2; range, 0–6 times) depending on the individual radio-oncological treatment concept.

A small part of patients (*n* = 109, 20,9%) remained hospitalized during the whole oncological therapy.

PICC was removed at accomplishing the treatment or in the case of a relevant complication approved by a treating radio-oncologist.

### Statistical analysis

Both groups of individuals treated as in- and outpatient were compared by means of univariate and multivariate analysis. The χ^2^test or Fisher’s exact test was used to compare categorical variables; Mann-Whitney U test was used to compare continuous variables. The multivariate analysis was performed as a regression analysis of the generalized linear models.

All *P* values were two-tailed, and *P* < 0.05 was considered statistically significant.

The analysis of complication rates in the in- and outpatient group was carried out as a binary logistic regression analysis using a generalized estimation equation, adjusted for insertion time. All analyses were performed using SPSS (Version 20.0).

## Results

The overall technical success rate of PICC insertion was 98.7%. A total of 522 PICC systems were used for data evaluation in the follow-up period. The baseline characteristics of the patients included are described in Table 1. The total indwelling time was documented to be 18,292 catheter days. The cumulative follow-up during hospitalization was 7954 days (43.5%) and 10,338 days (56.5%) during ambulant treatment. During the follow-up period, a total of 75 PICC-associated complications resulting in removal of the catheter were documented. The PICCs were mainly used for the application of chemotherapy (*n* = 360; 69%), immunotherapy (*n* = 31; 5.9%), parenteral nutrition (*n* = 42; 8.0%), intensified supporting care (*n* = 22; 4.2%) and combination of whose (*n* = 67; 12.9%). Table 2 shows complication rate depending on type of use.

**Table 1:** Characteristics of the patients studied

Characteristic	Number (%)
*Sex*	
Male	311 (59.6%)
Female	211 (40.4%)
*Age (years)*	
18–39	16 (3.1%)
40–65	204 (39.1%)
65+	302 (57.8%)
*ICD-10 diagnoses*	
C00–C14 lips, oral cavity, pharynx	86 (16.5%)
C15–C26 digestive organs	265 (50.8%)
C30–C39 respiratory system and intrathoracic organs	47 (9.0%)
C43–C44 skin	2 (0.4%)
C50 mammary gland	2 (0.4%)
C51–C58 female genitalia	59 (11.3%)
C64–C68 urinary organs	6 (1.1%)
C69–C72 central nervous system, eyes	6 (1.1%)
C73–C75 thyroid and other endocrine glands	2 (0.4%)
C76–C80 malignant neoplasms of ill-defined, other secondary and unspecified sites	45 (8.6%)
C81–C96 malignant neoplasms of lymphoid, haematopoietic and related tissue	2 (0.4%)
ECOG – performance status*	
0	281 (53.8%)
1	208 (39.8%)
2	28 (5.4%)
3	4 (0.8%)
4	1 (0.2%)
T-stage**	
1	55 (10.5%)
2	107 (20.5%)
3	193 (37.0%)
4	119 (22.8%)
N/S	48 (9.2%)
N-stage***	
0	131 (25.1%)
1+	333 (63.8%)
N/S	58 (11.1%)
*Treatment strategy*	
Neoadjuvant	150 (28.7%)
Adjuvant	199 (38.1%)
Definitive	173 (33.1%)
Intent-to-treat****	
Curative	395 (75.7%)
Palliative	127 (24.3%)

*ECOG performance status: Activity status to estimate general condition of oncological patients. The scale ranges from 0 (unimpaired activity) to 5 (death) [40]

**T-stage (tumour): extension and behaviour of the primary tumour

***N-stage (nodule): lack or presence of regional lymph node metastases

****Initial intent to treat, without consideration of the actual outcome

**Table 2:** Complication frequency in relation to use

Use	Frequency of use	Complication rate
Exclusively for chemotherapy	360 (69%)	38 (7.3%)
Exclusively for immunotherapy	31 (5.9%)	12 (2.3%)
Exclusively for parenteral feeding	42 (8.0%)	8 (1.5%)
Chemotherapy and parenteral feeding	51 (9.8%)	9 (1.7%)
Immunotherapy and parenteral feeding	16 (3.1%)	3 (0.6%)
Other	22 (4.2%)	5 (1.0%)
Overall	522	75 (14.4%)

The overall complication rate was 4.1 per 1000 catheter days.

The complication rate in the outpatient group was 3.6 per 1000 catheter days vs. 4.8 per 1000 catheter days in inpatient group. There was no difference in the number of complications in these two cohorts (OR 0.976; 95% CI [0.598; 1.619]; *p* = 0.924).

**Fig. 1:**
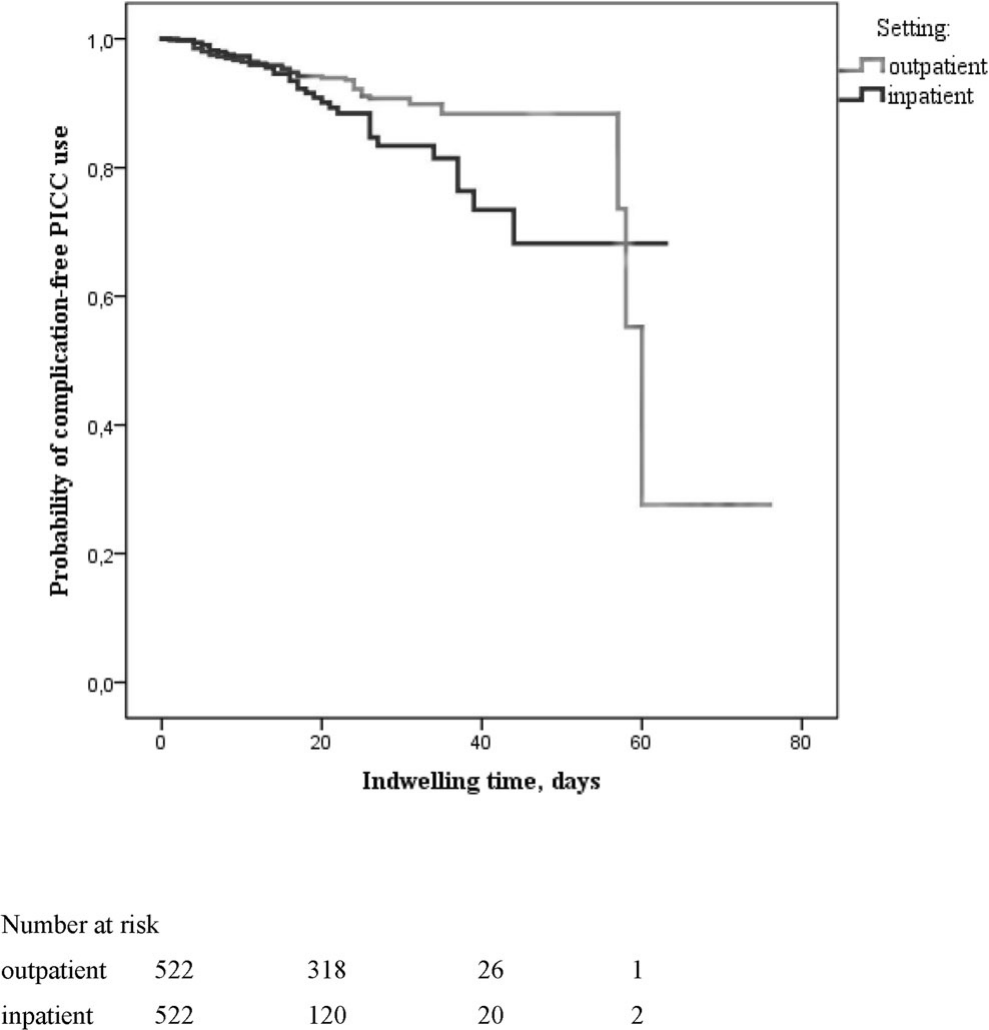
Likelihood of a complication-free PICC use

The median indwelling time of PICC was 37 days (1–97). By day 35, about 88.3% of PICCs were still in use by outpatients had no complications. About 81.5% of the actively used catheters in the inpatient group were still free of complications after 34 days of indwelling time (Fig. 1). As shown in Table 3, the most frequent observed complications were local inflammation at the insertion site (*n* = 23; 4.4%), venous thrombosis (*n* = 15; 2.9%), bloodstream infection (*n* = 11; 2.1%) as well as occlusion (*n* = 11; 2.1%) of the PICC.

**Table 3:** Types of complications and their frequency

Type of complication	Total frequency n (%) [per 1000 catheter days]	Outpatient frequency n (%) [per 1000 catheter days]	Inpatient frequency n (%) [per 1000 catheter days]
Local inflammation	23 (4.4%) [1.26]	13 (2.5%) [1.26]	10 (1.9%) [1.26]
Bloodstream infection	11 (2.1%) [0.60]	2 (0.4%) [0.19]	9 (1.7%) [1.13]
Occlusion	11 (2.1%) [0.60]	6 (1.1%) [0.58]	5 (1.0%) [0.63]
Dislocation	6 (1.1%) [0.33]	2 (0.4%) [0.19]	4 (0.8%) [0.50]
Deep vein thrombosis	15 (2.9%) [0.82]	9 (1.7%) [0.87]	6 (1.1%) [0.75]
Local bleeding	1 (0.2%) [0.05]	0	1 (0.2%) [0.13]
Septic thrombosis	2 (0.4%) [0.11]	1 (0.2%) [0.10]	1 (0.2%) [0.13]
Pulmonary artery embolism	1 (0.2%) [0.05]	1 (0.2%) [0.10]	0
Local allergic reaction	1 (0.2%) [0.05]	1 (0.2%) [0.10]	0
Accidental dislocation	4 (0.8%) [0.22]	2 (0.4%) [0.19]	2 (0.4%) [0.25]
Overall	75 (14.4%) [4.10]	37 (7.1%) [3.58]	38 (7.3%) [4.78]

Most of the PICC complications (*n* = 61, 11, 7%) were considered as minor adverse events, which resulted solely in venous access removal. Major adverse events (*n* = 14, 2.7%) leading to (prolonged) hospitalization were mostly due to PICC-associated bloodstream infection. They occurred at a rate of 0.7 per 1000 catheter days. Microorganism isolated from the PICC tip, as well as *S. aureus* bacteraemia, could be confirmed in 2 (0.4%) of 13 (2.5%) cases. In other cases, patients presenting septic symptoms had negative microbiological blood samples. One case was diagnosed with a pulmonary artery embolism.

In a multivariate analysis, only immunotherapy could be identified as an independent risk factor for complications (OR 5.6; 95% CI [2.4; 13.1]; *p* < 0.001).

## Discussion

Many cancer patients receiving radiotherapy require central venous access. The choice of the device is usually made by treating physician on the basis of medical criteria, e.g. kind of intended application and duration, availability, patient’s condition and guidelines.

So far, the assortment of available central venous devices for radiotherapy is limited in Germany. Currently, non-tunnelled CVCs are only used in hospitalized patients and ports are often preferred in outpatient care. The tunnelled CVC are less common central venous access types. They have low level of evidence in cancer patients receiving radiotherapy. The majority of tunnelled CVC types have been designed for highly specific medical applications (e.g. Demers® catheters for dialysis). Broviac® and Hickman® catheters are widely used in paediatric patients, whereas Groshong® and Broviac® catheters are common in adults undergoing haematopoietic stem cell transplantation or for home parenteral nutrition [20].

The use of non-tunnelled CVC as short-term access is limited to hospitalized patients and cannot be utilized in outpatient setting. It should stay in situ for no longer than 14 days to avoid complications [13, 14]*.* We would like express our fundamental misgiving in relation to frequent CVC placements because of insertion risk and patients stress.

Implantable port (chest-port, groin-port or upper-limb-port (PICC-port)) is suggested as permanent venous access requiring a minor surgery for placement. They are not appropriate for use < 30 days [13]. There is mostly no compelling need for them during standard radiation oncology protocols < 60 days either. The indication for the port system in the context of long-term multimodal therapy concepts should be made individually. In this regard, the PICC can be deployed with much more temporal flexibility, from a few weeks up to 6 months [2, 3, 14, 15].

A significant advantage of PICC over the port and tunnelled CVC is the low risk of procedure-related trauma, low risk of significant bleeding and easy to achieve local haemostasis even in a state of poor coagulation [15, 21].

The maintenance of PICC is simple; it requires only a professional competence in dealing with central venous catheters but no special training. Even a proportion of patients can be responsible for their own PICC care when discharged from hospital in between treatment regimens. The maintenance procedure is not demanding and stress-free because it is not associated with an invasive port chamber puncture [12].

The PICC removal is simple and can be quickly performed even by nurses. On the contrary, tunnelled CVC and port-related complications require a surgery for revision or explantation [22, 23].

PICC has many decisive advantages over other venous access types, but it has not been established on a nationwide basis because of various arguable reasons until now.

The German Commission for Hospital Hygiene and Infection Prevention [Kommission für Krankenhaushygiene und Infektionsprävention, KRINKO] negatively appraises PICCs as the preferred central venous access. These concerns arise from studies conducted on critically ill patients on ICU wards [24–27]. The insights however are probably not valid in other (e.g. cancer) patient groups.

One of the most frequent, clinically relevant complications of all types of intravascular devices (IVD) is bloodstream infections (BSI). The incidence rates of IVD-related BSI are lowest with ports (0.1–0.2 per 1000 catheter-days), the highest with non-tunnelled CVCs (2.3–2.7 per 1000 catheter days); PICCs show an intermediate risk profile (0.4–2.1 per 1000 catheter days) [24, 28, 29].

Irrespective the site of port placement, chest or upper limb, there is an identical risk of infectious complications [30]. The incidence of BSI in tunnelled CVC (1,8–7,9 per 1000 catheter days) is highly dependent on study population and CVC usage [31]. The highest complications were observed in immunocompromised hosts.

In our cohort, the overall rate of PICC bloodstream infections was 0.6 per 1000 catheter days, thus comparable to the rate reported previously.

Consistent with observations by Maki et al., bloodstream infections occurred less frequently 0.2 per 1000 catheter days in the outpatient setting, than in the hospitalized patients 1.2 per 1000 catheter days [24]. A lower risk of BSI in ambulant patients can be due a less frequent and in general different use of IVD. It suggests a favourable safety profile of PICC for the outpatient setting. However, the assessment of a bloodstream infection was determined clinically. In case of suspected BSI, the PICC was removed immediately. Most of the investigated blood cultures and catheter tips remained without microbiological pathogen detection. We can report detection of *Staphylococcus aureus* in two blood cultures.

The incidence of a local or systemic catheter-related thrombotic complication depends upon the IVD type, insertion site, diameter of the vessel, thrombophilia, criteria for diagnosis, etc. The highest reported incidence of thrombosis was therefore observed in critically ill patients, in individuals with prior thrombotic events, active tumour disease and systemic inflammation accompanied by leucocytosis >12 Gpt/l [7, 32–34]. These risk factors were present in our cancer patients to certain extent as well. We did not perform thrombosis prophylaxis routinely.

The risk of thrombotic event occurrence varies amongst different cancer entities. The highest rates have been observed in malignancies of the pancreas, brain and stomach [35, 36]. Kidney, uterus, bladder, lung, colon [35] as well as hematologic malignancies are associated with relatively high risk of thrombosis [37]. Lower risk of thrombotic event is suspected for patients with breast, head and neck or prostate cancer [37, 38]. Especially in the treatment of lung, mediastinal, head and neck cancer, the radiation field can involve the PICC and the corresponding venous vessel. Apparently the radiation beam itself can have thrombogenic effects on vessels [39]. It is not clear whether the accumulation of local thrombogenic factors in this patient group results in a higher rate of PICC-associated thrombosis.

A significant part of PICCs in our cohort (*n* = 237, 45,4%) was anatomically involved in to the radiation field (“head and neck cancer” *n* = 132; “oesophageal tumours” *n* = 77, “lung cancer/thoracic tumors” *n* = 28). This circumstance did not statistically increase the rate of local thrombotic events (*p* = 0,722). The greatest part (*n* = 10) of PICCs removed due to venous thrombosis was not situated in the radiation beam. The thrombogenic effect of the radiation to the venous vessel may be low and thus statistically not apparent. In our opinion, no change of clinical routine in placing PICCs in regard to a possible radiation field is needed.

In previous studies, PICC placement was associated with a greater risk of deep vein thrombosis (DVT) (OR 0.43; 95% CI 0.23–0.80) than ports 0.11 per 1000 catheter days [7, 28, 40]. The incidence of thrombotic complication for PICC varies between 5 and 15% for hospitalized patients and between 2 and 5% for ambulatory patients [41].

The incidence of thrombosis observed in our patients 2.8% (0.82 per 1000 catheter days) corresponds to that evidence.

As with all centrally inserted catheters, the incidence of thrombosis increases depending on the effective catheter diameter and thus the number of lumens used [7, 32, 42]. Double lumen PICCs were fully sufficient in our radio-oncologic patients; we routinely used only this PICC type.

As DVT is common in PICC, benefit risk profile assessment should be performed individually. The clinical situation and the patient’s needs should be examined and considered to provide an optimal therapy on the one hand but also minimize the risk of thrombosis on the other hand.

The most cases of PICC-associated DVT diagnosed clinically due to lacking duplex sonographic verification, which was not logistically available. One prospective study found that 75% of patients identified with DVT were asymptomatic [43]. That is why a higher thrombosis incidence in our cohort can be suspected. The prognostic meaning and the outcome of such occult undiagnosed DVTs remain unclear. Although the PICC does not necessarily have to be removed when detecting a thrombosis, we never left it in situ if this complication was suspected. Ultimately, it could have continued to be used if patency and no infection were assumed [44].

In our analysis, multivariate logistic regression found the immunotherapy with cetuximab and was an independent risk factor for PICC-related complications. Out of 31 patients who received the immunotherapy, 9 developed inflammation at the catheter exit site. Common side effects with cetuximab include disseminated erythema, acneiform rashes and local superinfections [45]. These cutaneous side effects are mediated via the EGF receptor of the skin and hair follicles. It is possible that the increased incidence of local skin reactions at the PICC exit site in patients receiving cetuximab therapy can be explained by this aetiological mechanism. The recommendations for the management of cutaneous side effects of immunotherapy are not designed to address local complications at the catheter exit sites and are, therefore, not transferable to them.

The therapeutic regimen of thoracic, ENT (ear-nose-throat) and mediastinal cancer patients regularly include the irradiation of the supraclavicular or cervical lymph node regions in combination with systemically applied chemotherapy or immunotherapy. CVCs and ports inserted into the jugular and subclavian veins, as well as associated dressings can interfere with the irradiation fields. Other possible complication is a pronounced radiodermatitis with skin desquamation and superinfection. In such situations, a PICC or PICC-port placed on the upper arm, hence outside the irradiation field, is a very good alternative to manage the treatment successfully.

The main limitation of our study is not only the retrospective data assessment but also the subjectivity of clinical decisions made in particular patient. All patients were treated by a doctor team. The amount of documented complications, the appraisal of their severity and particular their management were dependent on the percepted clinical context, doctor’s qualification and expertise, as well the on patient factors. Wishing to minimize PICC associated risks especially in ambulant patients, we removed the culprit IVD on a suspicion of complication generously. This corresponds to a very conservative and defensive PICC management, which leads to statistically elevated complication rate. To realize a more liberal management, a practical experience gain in dealing with the PICC on both the medical and nursing sides (nursing services, general practitioners) is needed.

In summary, PICCs represent a safe alternative to the port system implantation in cancer patients receiving radiotherapy and maintaining the venous access for repeated admissions on out- or inpatient basis from weeks to a maximum of 6 months.

## Electronic supplementary material

ESM 1 (PNG 1896 kb)

High Resolution image (TIFF 5321 kb)
