# Supplementary figures and images for: Peripherally inserted central venous catheter (PICC) in outpatient and inpatient oncological treatment
Source: Support Care Cancer. 2020 Jan 22;28(10):4753–60. doi: 10.1007/s00520-019-05276-0 (PMC7447660; doi:10.1007/s00520-019-05276-0)

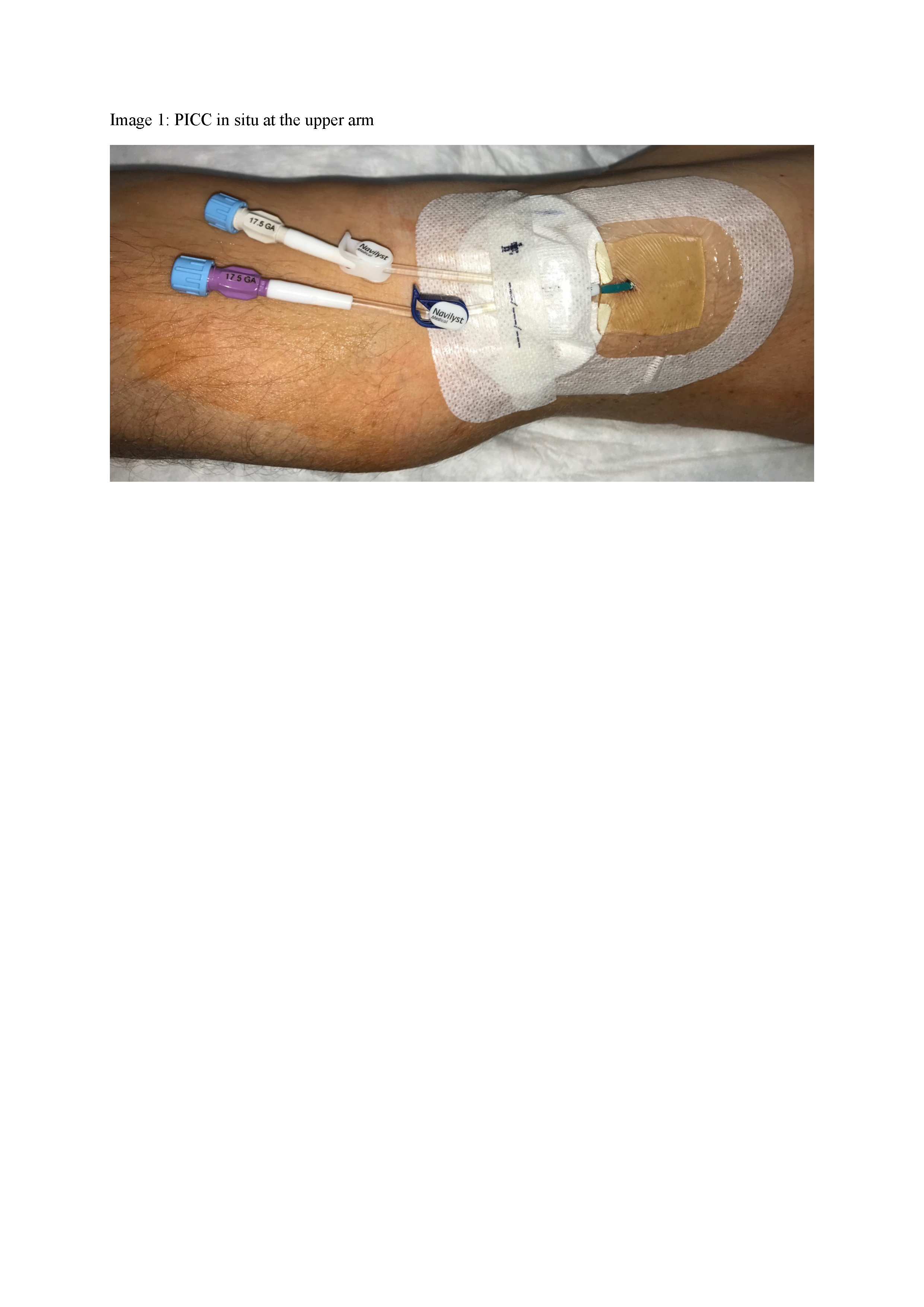

Supplement: Supplementary file 1 — (PNG 1896 kb) [file 520_2019_5276_FIG2_ESM.png]

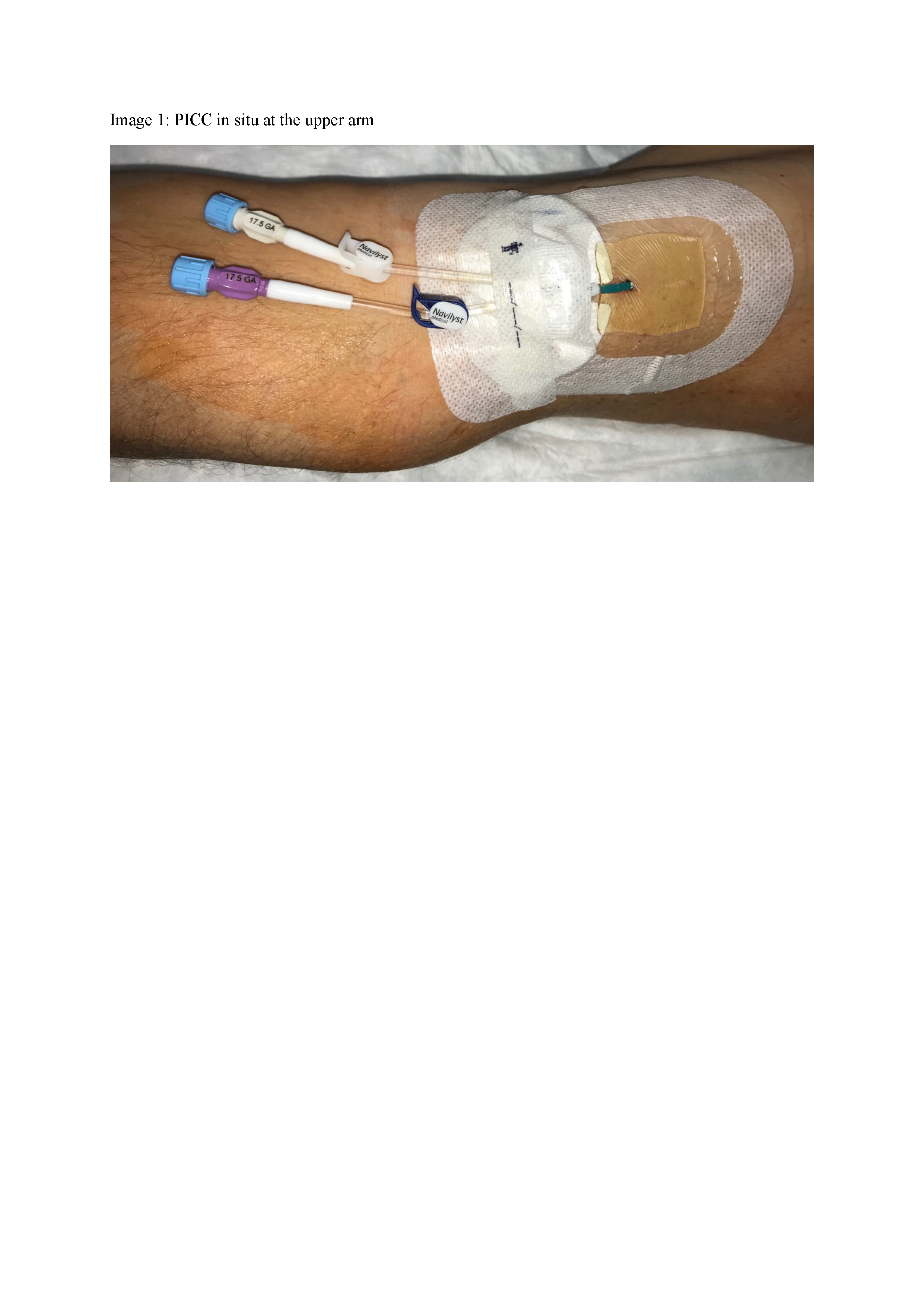

Supplement: Supplementary file 2 — High Resolution image (TIFF 5321 kb) [file 520_2019_5276_MOESM1_ESM.tiff]
